# Rare and common manifestation of musculoskeletal and sinonasal sarcoidosis: A case report

**DOI:** 10.1016/j.radcr.2024.05.032

**Published:** 2024-06-08

**Authors:** Kihyun Kwon, Eric Taub, Brandon Dang, Joseph Dobtsis

**Affiliations:** Harlem Hospital Center/Columbia University Irving Medical Center, 506 Lenox Ave, New York, NY 10037, USA

**Keywords:** Sarcoid arthropathy, Osseous sarcoid, Systemic sarcoid, Sarcoid rhinosinusitis, Sinonasal sarcoidosis

## Abstract

Sarcoidosis is a systemic granulomatous disease that can affect multiple organ systems. Although many sarcoidosis patients are asymptomatic, the variable clinical progression of symptomatic patients and the nonspecific presentation make diagnosis difficult in certain cases. Musculoskeletal and sinonasal involvement of sarcoidosis are uncommon manifestations, and they are often only seen in patients with widespread disease. Diagnosis of osseous sarcoidosis, sarcoid arthropathy, and sarcoid rhinosinusitis are typically based on a combination of clinical history, radiological findings, and pathologic specimens. Although there are classic image findings, such as lacelike honeycomb appearance of small bones of the hands or hilar/mediastinal lymphadenopathy, sole reliance on image findings for the diagnosis of sarcoidosis is unreasonable as many findings are nonspecific. However, failure to include sarcoidosis in the differential diagnosis often leads to a delay in recognition of musculoskeletal or sinonasal involvement and results in ineffective treatment plan. Even in patients with biopsy-proven sarcoidosis, some image findings in isolation that may represent granulomatous infiltrates are disregarded as nonspecific without raising the possibility of sarcoidosis due to its rare occurrence. Here we discuss a case of multisystemic sarcoidosis in a 42-year-old female with a constellation of classic and rare findings of biopsy-proven sarcoidosis.

## Introduction

Sarcoidosis is a systemic, granulomatous disease of unknown pathogenesis [1]. The exact cause is unknown and a wide range of speculative hypotheses have been brought up. Clinical and histological similarities with mycobacterial and fungal disease have suggested infection as a plausible etiology while recent studies suggest evidence of a genetic component implicated in susceptibility to sarcoidosis [2]. Manifestation is variable due to its tendency to involve multiple systems. Clinical signs and symptoms are often nonspecific, and patients may present with fatigue, weight loss, or general malaise; approximately 50% of patients remain asymptomatic [4]. Despite thoracic sarcoidosis and bilateral hilar lymphadenopathy being the most common findings, extra-thoracic involvements are the initial manifestation in 50% of symptomatic patients [1,4].

**Fig. 1:**
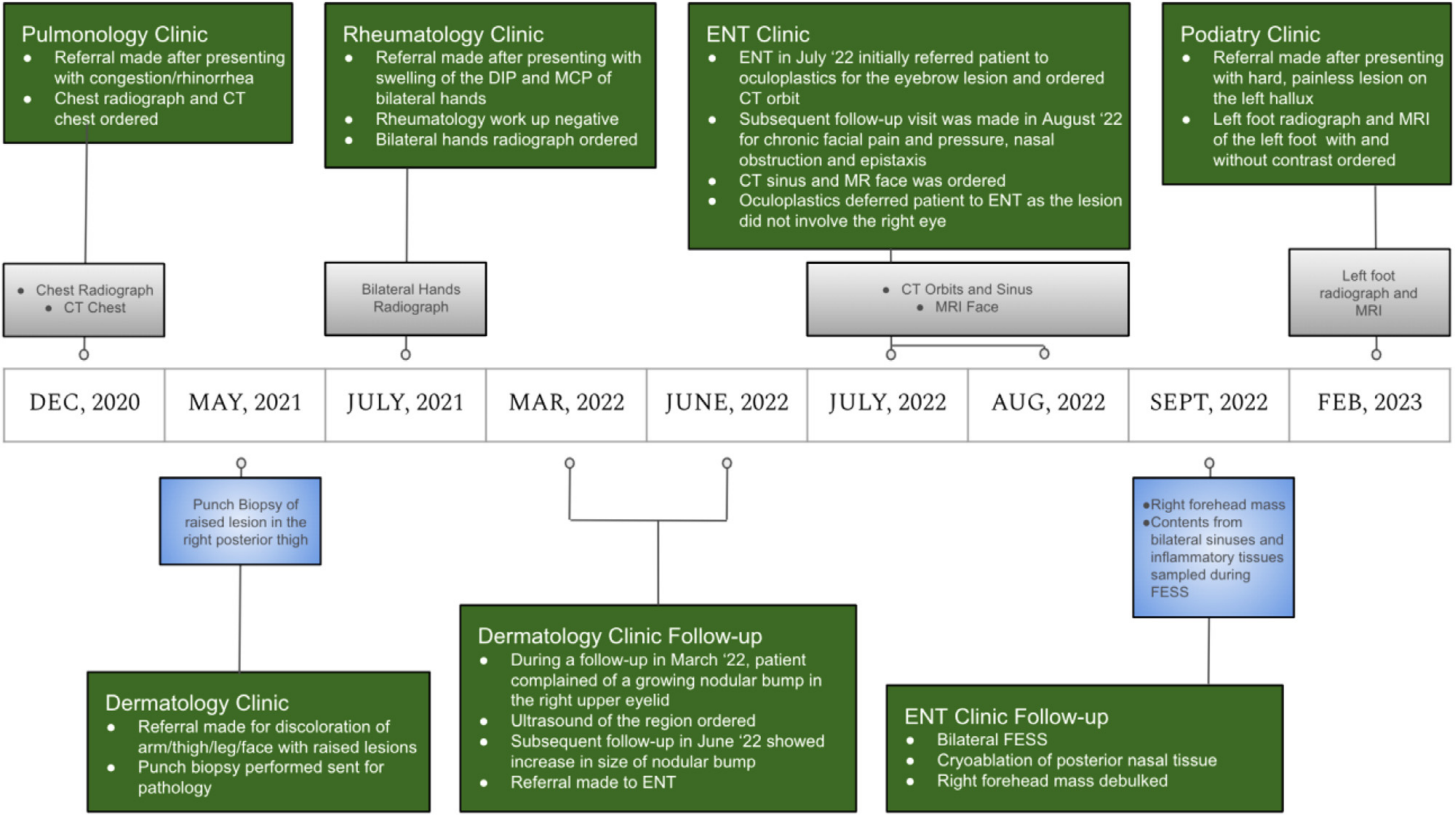
Timeline of patient's different clinic visits, radiologic images performed, and pathologic tissue samples to confirm diagnosis of sarcoidosis.

Typical sarcoidosis patients are young or middle-aged adults between 25 and 45 years old with a peak incidence between 20 and 29 years old [1,5]. The disease has distinct geographic and racial predilections; in the United States, sarcoidosis is more prevalent in African Americans compared to Caucasians [9] whereas Swedes and Danes are also commonly affected by sarcoidosis. There is also a slightly higher prevalence in women [4]. Clinical progression in patients with sarcoidosis is variable with two-thirds of patients achieving spontaneous remission, and 10%-30% of patients progressing to chronic disease [1]; clinical progression and prognosis may also correlate with the mode of onset where an acute onset with erythema nodosum is more likely to result in self-limiting course with spontaneous resolution compared to an insidious onset with multiple extrapulmonary lesions [4]. There have been suggestions that subjects with black skin have a more severe form of the disease [1]. Our patient is a middle-aged female with dark skin complexion who presented with a myriad of extrapulmonary symptoms and multiorgan involvement that prompted an assortment of specialty visits, radiologic imaging, and pathology diagnosis (Fig. 1).

## Case report

**Fig. 2:**
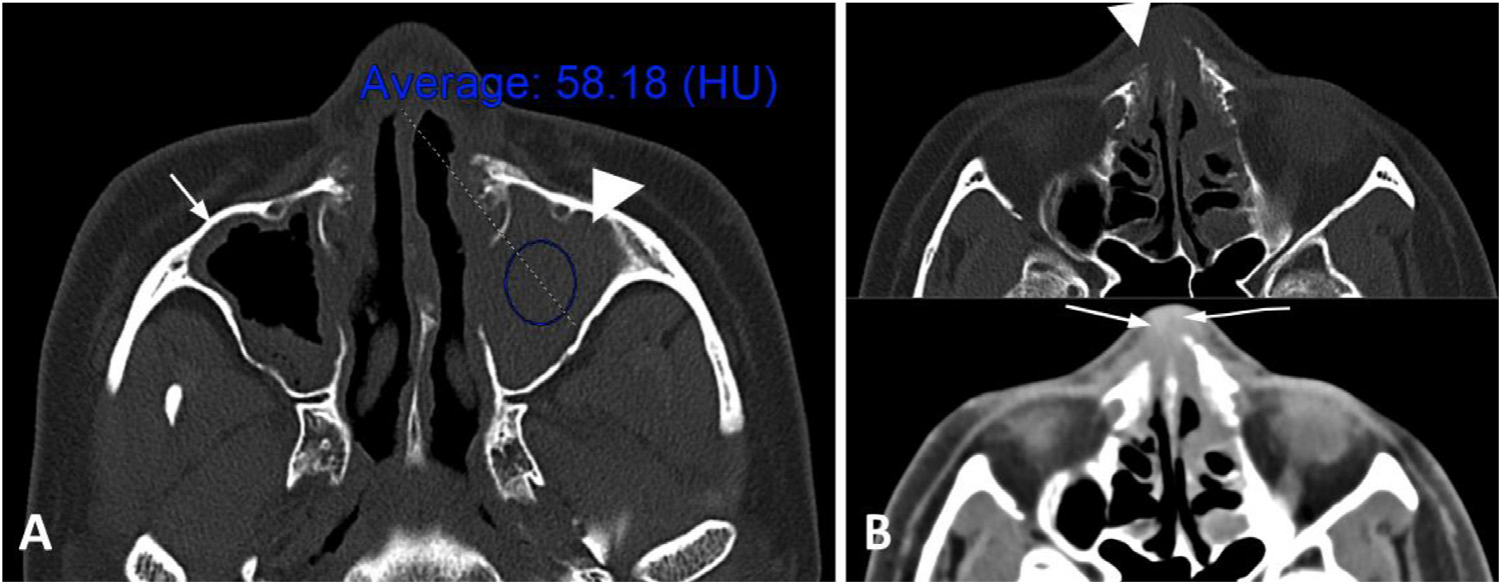
(A) Axial CT of the sinuses without contrast demonstrates complete opacification of the left maxillary sinus (white arrowhead), mucosal thickening of the right maxillary sinus (white arrow). The fluid contents of the left maxillary sinus measured average 58 HU, representing inspissated secretions. **(**B) Axial CT of the sinuses without contrast in bone and soft tissue window at the level of the ethmoid air cells demonstrates partial opacification of the ethmoid air cells. There is a soft tissue density along the nasal bridge (white arrow) with partial erosion and flaring of the nasal bones (white arrowhead).

**Fig. 3:**
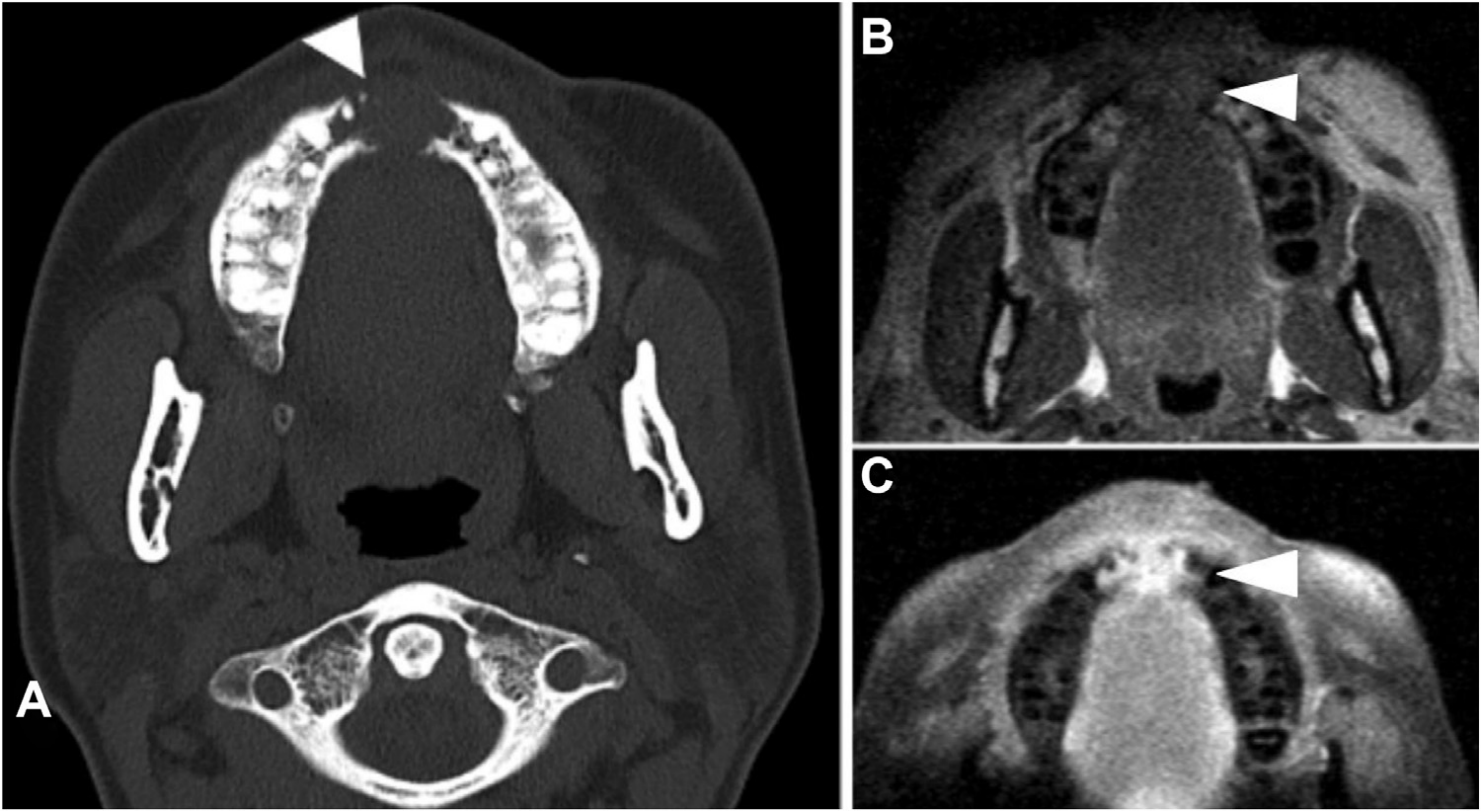
(A**)** Axial CT of the sinus without contrast demonstrates an erosive mass within the anterior aspect of the lower maxilla (white arrowhead). (B and C) Pre and Postcontrast Axial T1 images show an enhancing T1 isointense mass in the anterior lower maxilla (white arrowheads).

**Fig. 4:**
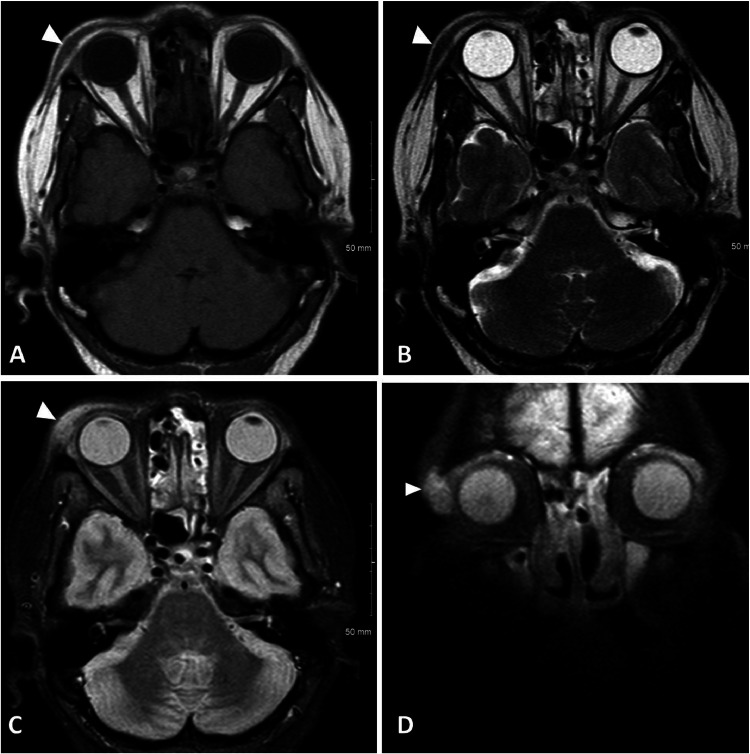
(A) Axial T1 of the face shows a T1 hypointense spindle shaped soft tissue mass in the right periorbital soft tissues (white arrowhead). (B**)** Axial T2 image of the face shows T2 hypointensity of the respective mass (white arrowhead). (C and D) Axial and Coronal T1 post contrast images of the face show enhancement of the mass (white arrowhead).

**Fig. 5:**
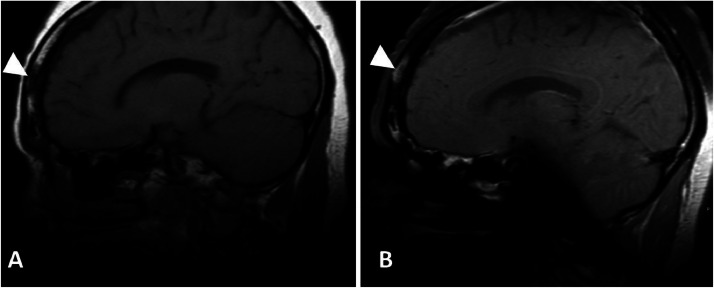
(A and B) T1 pre and post contrast sagittal images of the head demonstrates an enhancing T1 hypointense lesion in the frontal bone (white arrowheads).

The patient is a 42-year-old female born in Trinidad and Tobago who presented to the general clinic with numerous complaints, including facial skin lesions, congestion, rhinorrhea, hand joint tenderness, and raised lesions in the extremities. The patient was referred to dermatology, pulmonology, and rheumatology for the symptoms. At the dermatology clinic, physical examination showed bilateral face pale discoloration, red/purple hyperpigmented nodules, and plaques involving the cheeks and nose, hypopigmented patches in the upper legs, and raised lesions within the hypopigmentation with pus-like drainage material. Punch biopsy was performed on a lesion in the right posterior thigh, and the pathology report described non-necrotizing epithelioid granulomas in the dermis with a diagnosis of “granulomatous dermatitis, consistent with sarcoid”. On a dermatology follow-up 10 months after the punch biopsy, the patient complained of a hard, mobile nodular lesion in the temporal aspect of the right supraorbital bone near the right eyebrow that was not present on the initial visit. The lesion demonstrated interval growth on a 3-month follow-up, and the patient also noted occasional discomfort and tenderness to palpation. The patient was referred to ENT after ultrasound of the lesion and CT of the orbits were performed. Moreover, the patient also complained of chronic facial pain and pressure, nasal obstruction, and purulent discharge associated with epistaxis, which prompted further imaging with CT of the sinus and MRI of the face (Fig. 2, Fig. 3, Fig. 4, Fig. 5). The images from the CT show dense opacification within the maxillary sinuses and ethmoid air cells suggestive of inspissated secretions, and osteolytic soft tissue lesions within the anterior aspect of the lower maxilla and nasal bridge (Fig. 2) [3]. MRI images provide additional information: enhancing soft tissue lesion within the lower maxilla and a soft tissue mass in the right periorbital region that demonstrates T1 and T2 hypointense signal and contrast enhancement (Fig. 4). There is also a T1 hypointense, enhancing osseous lesion in the frontal calvarium (Fig. 5).

ENT decided to plan for full bilateral functional endoscopic sinus surgery (FESS) and excision of the right forehead lesion. According to the ENT intra-operative note, the right forehead mass had no discrete planes around the mass, and the tissue was highly infiltrative. The mass was debulked, and the excised mass was ultimately sent for pathologic analysis. Additionally, contents from bilateral sinuses and inflammatory tissues at the level of the anterior nasal floor were sampled during FESS and also sent to pathology. Both the right forehead mass and samples from FESS showed extensive non-caseating granulomas consistent with sarcoidosis.

**Fig. 6:**
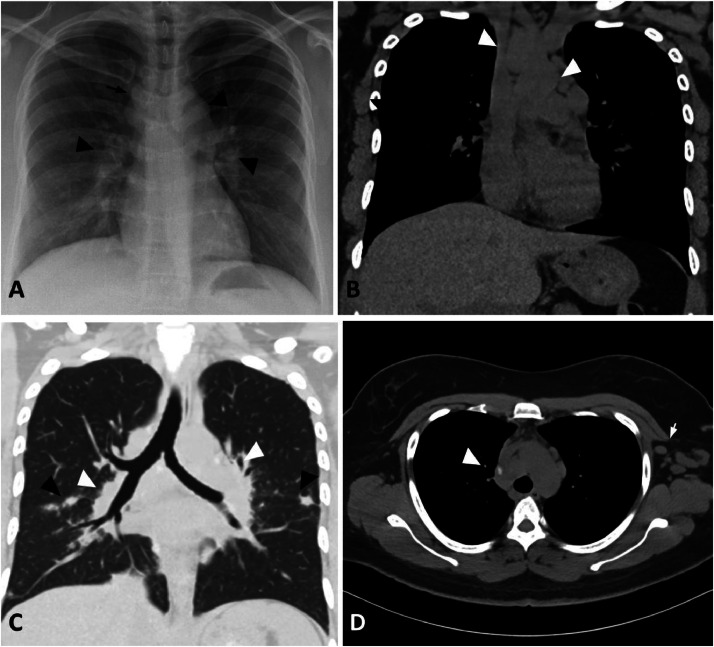
(A) Posteroanterior Radiograph of the chest shows thickening of the right paratracheal stripe (black arrow) with lobular contour of the aortic knob and lung hila (black arrow heads) representing mediastinal and hilar adenopathy. (B) Coronal CT image of the chest without contrast in soft tissue window at the level of the mid-axilla demonstrates prominent mediastinal lymph nodes (white arrowheads). (C) Coronal CT image of the chest without contrast in lung window at the level of the carina demonstrates multiple peri-fissural and subpleural nodules (black arrowheads) and hilar lymph nodes with adjacent central nodules (white arrowheads). (D) Axial CT image of the chest without contrast in soft tissue window at the level of the mid trachea shows calcified mediastinal lymph nodes (white arrowhead) and prominent axillary lymph nodes (white arrow).

Referral to pulmonology was made for rhinorrhea. Routine chest radiograph and subsequent high-resolution CT chest show diffuse axillary, mediastinal, and hilar lymphadenopathy and clusters of small central and subpleural lung nodules bilaterally, consistent with stage II sarcoidosis (Fig. 6).

**Fig. 7:**
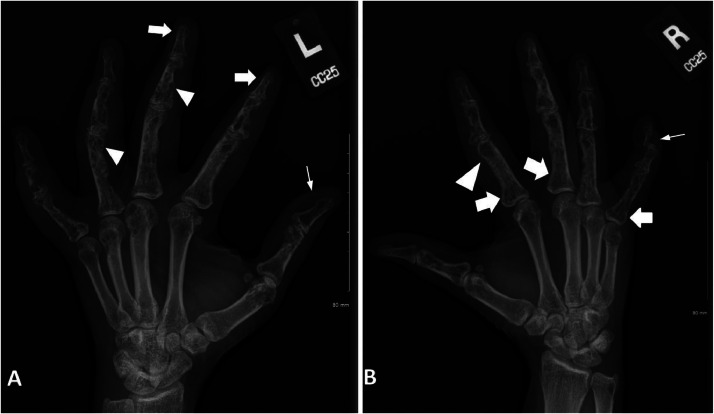
(A) Oblique radiograph of the left hand demonstrates multiple, well-delineated, small round lucencies throughout the phalanges (white arrowhead) with bony erosions predominantly in the first distal phalanx (white arrow) and second through fourth middle phalanges. “Lace-like” reticulation is noted at the tufts of the 2nd and 3rd phalanges (wide white arrows). Diffuse soft tissue swelling is noted along the digits. (B) Oblique radiograph of the right hand demonstrates similar findings of multifocal round lucencies with bony erosions predominantly affecting the fifth distal phalanx (white arrow). Note the solitary focal lucency in the distal ulna. Larger, bullous cyst-like lesion in the second distal phalanx (white arrowhead). Similar “lace-like” reticulation is noted at the base of multiple proximal phalanges (wide white arrows).

Following the punch biopsy, the patient was further worked up in the rheumatology clinic for joint tenderness and stiffness in the hands. At the clinic, the physical exam showed swelling of the distal interphalangeal and metacarpophalangeal joints of bilateral hands with limited mobility; dried pustules on the fourth and fifth metacarpals and second and third metatarsals were also visualized. Rheumatologic lab work-up was negative. Radiographs of the bilateral hands were obtained (Fig. 7), which show multifocal round lucencies throughout multiple phalanges with associated osseous erosion; some round lucencies are well-circumscribed, smaller, and clustered in groups, whereas few lucencies are larger in size. Additionally, along the tufts and base of the proximal phalanges, the radiographs show thickened trabeculae and thinning of the cortex resulting in a lace-like pattern of osteolysis.

**Fig. 8:**
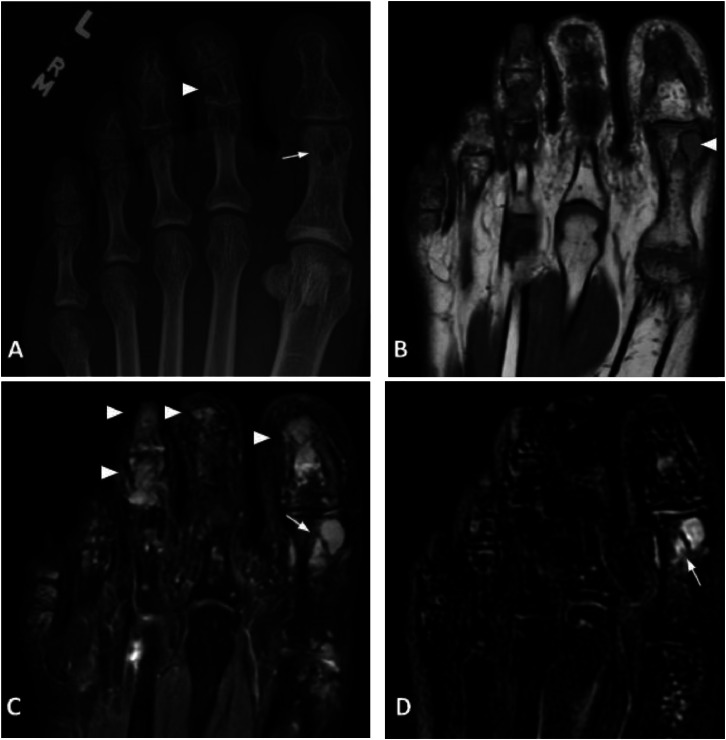
(A**)** AP Radiograph of the left foot demonstrates multifocal lucencies with erosive changes along the phalanges (white arrowhead). There is a prominent lucent lesion in the distal aspect of the proximal hallux (white arrow). (B) Axial T1 image of the left foot at the level of the first digit shows bilobed T1 hypointense lesion in the distal aspect of the first proximal phalanx (white arrow), corresponding with lucent lesion noted on prior radiograph. Additional T1 hypointense lesions (white arrowheads) are visualized in the second and third phalanges. (C) Axial STIR image of the left foot shows bilobed T2 hyperintense lesion in the first proximal phalanx (white arrow). Note additional areas of STIR hyperintensity in the first distal phalanx, middle and distal third phalanx, and distal fourth phalanx (white arrowheads). (D) Axial T1 postcontrast subtraction image of the left foot shows enhancement within the bilobed first proximal phalanx lesion (white arrow).

Few years after the initial diagnosis, the patient developed a hard lesion on her left hallux that presented with minimal pain when cutting her nails and minor bleeding. The patient was seen by a podiatrist. Physical examination of the foot showed a short trimmed left hallux nail with small, minimally raised tissue on the distal nail bed. Radiograph and MRI of the left foot were performed (Fig. 8). The radiograph of the left foot shows similar multifocal round lucencies with erosive changes in multiple phalanges as seen on radiograph of the hands. Numerous T2 hyperintense and T1 hypointense lesions on the MRI correspond to lucencies seen on the radiograph, including a larger, bilobed lesion in the proximal hallux demonstrating contrast enhancement.

## Discussion

Sarcoidosis is an entity that can present in various different ways; more often than not, the symptoms are nonspecific, and most patients are asymptomatic. As aforementioned, clinical progression is variable, and multiple organ systems can be involved in the pathogenesis. Similarly, imaging findings of sarcoidosis are relatively nonspecific and variable, and diagnosis with sole reliance on radiologic imaging may be difficult. Our patient had a myriad of chief complaints involving several organ systems resulting in numerous imaging studies. Pathology-proven diagnosis of sarcoidosis prompted attentive evaluation of the radiologic findings as the patient's images demonstrated rare sinonasal and musculoskeletal involvement in addition to classic imaging findings on chest radiograph and chest CT (Fig. 6).

Musculoskeletal manifestation in sarcoidosis patients is uncommon, affecting approximately 25%-33% of patients [3]. Osseous involvement is a rare occurrence in that subset of patients; 3%-13%, and an average of 5%, of sarcoidosis patients are diagnosed with osseous sarcoidosis [3,11]. However, as only 50% of patients with osseous sarcoidosis present with symptoms, an assumption can be made that osseous sarcoidosis are more common than reported [12]. Osseous involvement occurs much later in the disease course after it has progressed to an advanced stage, and patients with osseous sarcoidosis typically encounter 3 or more organ involvement compared to those without osseous involvement [1,3]. Small bone sarcoid lesions of the hand or foot are seen in 5%-7% of sarcoidosis patients, particularly in the middle and distal phalanges of the second and third digits; they are usually bilateral and asymmetrical [1]. Osseous sarcoid lesions are thought to be caused by granulomatous lesions infiltrating the Haversian canal in the perivascular space and resultant cortical bone destruction [1]. Patients are frequently asymptomatic; however, when the patients are symptomatic, the findings are nonspecific, ranging from sausage dactylitis to bone pain, and arthropathy [2,6]. In up to 40% of patients, musculoskeletal involvement often manifests as inflammatory arthralgia [4]. Most commonly affected joints in patients with sarcoid arthropathy are the knees, ankles, elbows, and wrists; however, abnormalities are rarely identified on radiographs [4]. In addition to the hand and foot involvement, our patient also complained of bilateral knee joint pain and stiffness; subsequent bilateral knee radiographs were negative.

Three types of osseous sarcoid lesions have been described on radiographs: type I is characterized by large, bullous cystic lesions that are often accompanied by pathologic fractures and rarely seen. Type II is typically described as multiple small, well-delineated, rounded cysts, whereas type III is characterized as “lace-like” or reticulated in appearance caused by tunneling of the cortex of the phalanx, which consequently leads to remodeling of the cortical and trabecular architecture [2]. Type III lesions are classic features of osseous sarcoidosis in small bones of the hand and feet, and they are also known as Perthes-Jȕngling's disease [3,5]. Our patient's radiographs of the bilateral hands (Fig. 7) display all three types of sarcoid lesions in multiple phalanges with numerous lucent lesions, some of which are small and clustered while some of which are larger and bullous. A lace-like reticular pattern is also evident in addition to a mixture of diffuse sclerotic changes and decreased mineralization.

Left foot radiograph (Fig. 8A) shows similar findings as seen on the bilateral hand radiographs. Further characterization with an MRI of the left foot (Figs. 8B–D) shows multiple T1 hypointense and T2 hyperintense lesions, some of which exhibit enhancement, osseous erosion, and trace intermetatarsal space bursitis. MRI tends to be nonspecific when it comes to musculoskeletal involvement of sarcoidosis. The appearance of osseous lesions on MRI is variable. As seen with this particular case, study by Moore et al. observed that most cases on MRI demonstrated lesions with decreased signal intensity on T1-weighted images and increased signal intensity on inversion-recovery, T2-weighted, and fat-saturated proton-density weighted images [6]. Benefits of MRI include showing invasion of the bone marrow, soft-tissue infiltration, and muscular lesions. Other nonspecific findings that may be associated with concomitant sarcoid arthropathy seen on MRI include tenosynovitis, tendinopathy, bursitis, and synovitis [5,6]. Additional advantage of performing an MRI is identifying subtle osseous lesions that are not visible on radiographs or CT.

MRI of the face (Fig. 3, Fig. 4, Fig. 5) provide details on the supraorbital soft tissue subcutaneous lesion that corresponds with a growing mass near the right eyebrow on physical exam. Osseous lesions involving the sinus and the calvarium are also well visualized on the MR images. Sarcoid lesions of the calvarium can be occult on radiographs and are considered uncommon to detect on plain radiographs [6]. Radiographs usually show osteolysis throughout the thickness of the cranial vault without peripheral sclerosis. The lesions are variable in size and sites are generally extensive [1]. If occult on radiograph, then they can be better depicted on MRI; however, the nonspecific finding has an appearance resembling that of an osseous metastatic lesion [6]. The osseous lesion in the frontal calvarium described in our patient's MRI is T1 hypointense and enhancing (Fig. 5), similar to the lesions characterized in the left foot MRI (Fig. 8). However, unlike the more commonly seen osseous involvement of the small bones in the hand and feet, sarcoidosis of the cranial vault is very rare and generally isolated [1]. Axial skeleton involvement is rarely reported; however, even within the spectrum of axial skeletal involvement, sarcoidosis more often affects the spine as opposed to the skull [5,13]. Involvement of the calvaria is widely underestimated due to lack of associated symptoms [1]; our patient's calvarial lesion was incidentally detected on the MRI while the patient was being followed for sinonasal symptoms and supraorbital soft tissue lesion.

Sinonasal symptoms were present when the patient first presented at the general clinic with congestion and rhinorrhea. After nearly 2 years, the patient's symptoms progressed to chronic facial pain/pressure, nasal obstruction, and purulent nasal discharge with occasional epistaxis. CT images (Fig. 2A) demonstrate inspissated opacification of multiple sinuses. The contents from the sinuses from bilateral FESS were sent to pathology and showed extensive non-caseating granuloma. In addition to the sinusoidal opacification, CT (Fig. 2B) images also show soft tissue thickening overlying the nasal bridge associated with erosion of the nasal bone and septum. Granulomatous involvement of sinonasal mucosa was described by Boeck in 1905, and sinonasal involvement in sarcoid patients is a rare manifestation, occurring approximately in between 0.7% and 6% of patients [8]. Due to its rarity and its nonspecific presentation, sinonasal sarcoidosis/sarcoid rhinosinusitis are often difficult to diagnose and treatments are delayed, which can result in patients suffering from chronic symptoms, mucosal damage, polyposis, nasal septal perforation, and repeated sinus surgeries [7]. Compared to conventional thoracic sarcoidosis, imaging features of sinonasal sarcoidosis have not been thoroughly scrutinized. A retrospective analysis of 22 biopsy-proven patients by Braun et al. demonstrated that the most commonly encountered CT findings were nodules of the turbinates and septum [8]. Our patient's CT does not demonstrate these findings. Similarly, the main MRI features of the only 3 biopsy-proven cases in the retrospective study were nodules on the septum and inferior turbinates; other MR findings included septal mucosal thickening and lysis of the septum [8].

Highly variable symptoms and nonspecific imaging findings in sarcoidosis patients make sarcoidosis a difficult diagnosis to make without pathology. Our patient had typical presentations of sarcoidosis, such as nasal bridge skin lesion classically described as lupus pernio facial rash, and classical image finding of hilar lymphadenopathy leading to prompt diagnosis via punch biopsy of a skin lesion. However, despite the early path-proven diagnosis, rare entities like sinonasal sarcoidosis or osseous sarcoid of the cranial vault were diagnosed almost 2 years after the initial clinic visit and over 1.5 years after the punch biopsy. Especially in patients with biopsy-proven sarcoidosis, there must be a high index of suspicion when patients have even the slightest symptoms, such as rhinorrhea or headache, and radiologic studies should be obtained. Radiologic images play a huge role despite the low specificity; imaging of sarcoidosis patients helps analyze the extent of the disease, identify subtle sarcoidosis lesions that are otherwise asymptomatic, and pinpoint plausible causes if symptomatic. As seen with our patient, cross-sectional studies helped identify the cause of the patient's sinonasal symptoms while also showing the extent of osseous involvement. Radiographs are effective when identifying classic findings such as hilar lymphadenopathy or “lace-like” appearance of phalanges. However, radiographs can be limited as they are often negative in symptomatic patients, and, if there are further clinical concerns in a patient with persistent or worsening symptoms, then advanced imaging modality should be utilized. Although it may be more difficult in the setting of isolated occurrence without symptomatic multiorgan involvement, incidental findings of osseous lesions or inspissated sinusitis in certain populations on imaging should include sarcoidosis as a differential diagnosis.

## Patient consent

Informed consent was obtained from the patient at 16:20 February 5, 2024. I explained to the patient what a case report is, what kind of information will be and will not be included in the case report, and which journal the article will be submitted to. Patient confidentiality was emphasized and conveyed to the patient. I also explained to the patient that images from his/her radiologic images will be used after removal of HPIs. Finally, I asked the patient if there were any concerns or questions, to which the patient replied “no”. Patient consented to the publication of the patient's case.
